# Supplementary material for: BMS-986158, a Small Molecule Inhibitor of the Bromodomain and Extraterminal Domain Proteins, in Patients with Selected Advanced Solid Tumors: Results from a Phase 1/2a Trial
Source: Cancers (Basel). 2022 Aug 23;14(17):4079. doi: 10.3390/cancers14174079 (PMC9454848; doi:10.3390/cancers14174079)
Supplement: Supplementary file 1 [file cancers-14-04079-s001.zip › cancers-1813612-supplementary.pdf]

cancers

MDPI

Supplemental Materials

Title: BMS-986158, a Small Molecule Inhibitor of the Bromodomain and Extraterminal Domain Proteins, in Patients With Selected Advanced Solid Tumors: Results From a Phase 1/2a Trial

John Hilton,[1] Mihaela Cristea,[2] Sophie Postel-Vinay,[3] Capucine Baldini,[3] Mark Voskoboynik,[4,5] William Edenfield,[6] Geoffrey I. Shapiro,[7] Michael L. Cheng,[7] Jacqueline Vuky,[8] Bradley Corr,[9] Sharmila Das,[10] Abraham Apfel,[10] Ke Xu,[10] Martin Kozicki,[10] Keziban Ünsal-Kaçmaz,[10] Amy Hammell,[10] Guan Wang,[10] Palanikumar Ravindran,[10] Georgia Kollia,[10] Oriana Esposito,[10] Shodeinde Coker,[10] Jennifer R. Diamond[9]

[1]Ottawa Hospital, Division of Medical Oncology, Ottawa, Canada; [2]City Of Hope National Medical Center, Department of Medical Oncology & Therapeutics Research, Duarte, CA; [3]Institut Gustave Roussy, Drug Development Department, Villejuif, France; [4]Alfred Health, Department of Medical Oncology, Melbourne, Australia; [5]Monash University, Central Clinical School, Melbourne, Australia; [6]Prisma Health Cancer Institute, Greenville, SC; [7]Dana-Farber Cancer Institute, Boston, MA; [8]Oregon Health & Science University, Portland, OR; [9]Univeristy of Colorado Anschutz Medical Campus, Aurora, CO; [10]Bristol Myers Squibb, Princeton, NJ

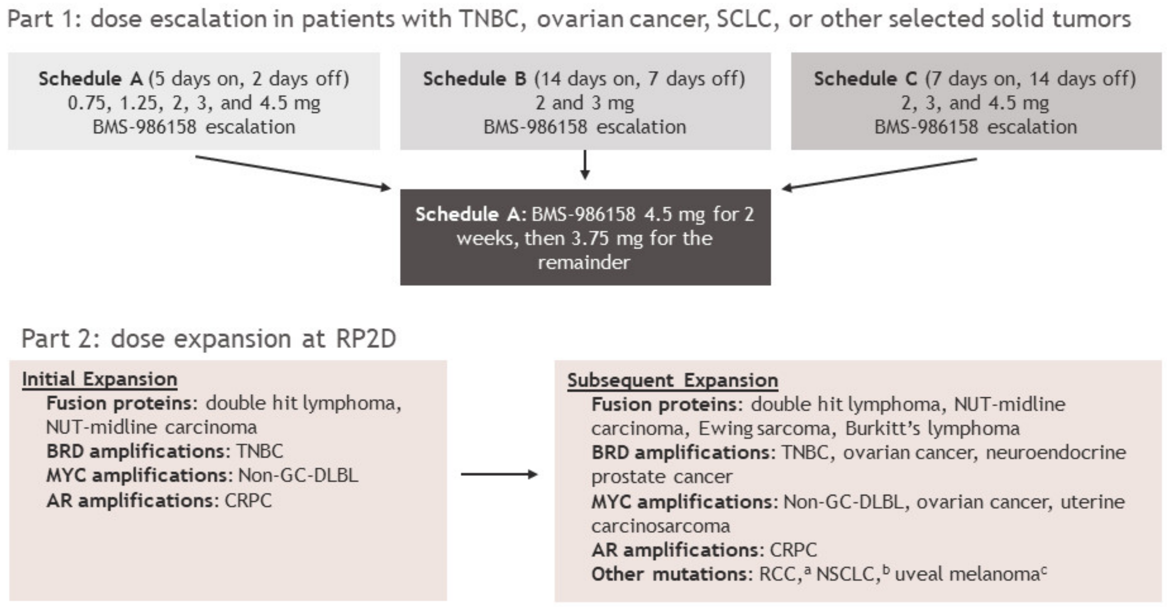

Figure S1. Study design. [a]Renal cell carcinoma with *SWI*/*SNF* mutations; [b]Non-small cell lung cancer with *SWI*/*SNF* or *KRAS*/wt*TK11*; [c]Uveal melanoma with *Gnaq*/*11*. AR, androgen receptor; BRD, bromodomain; CRPC, castration-resistant prostate cancer; Non-GC-DLBL, non-germinal center diffuse large B-cell lymphoma; NSCLC, non-small cell lung cancer; NUT, nuclear protein in testis; RCC, renal cell carcinoma; RP2D, recommended phase 2 dose; SCLC, small cell lung cancer; TNBC, triple-negative breast cancer.

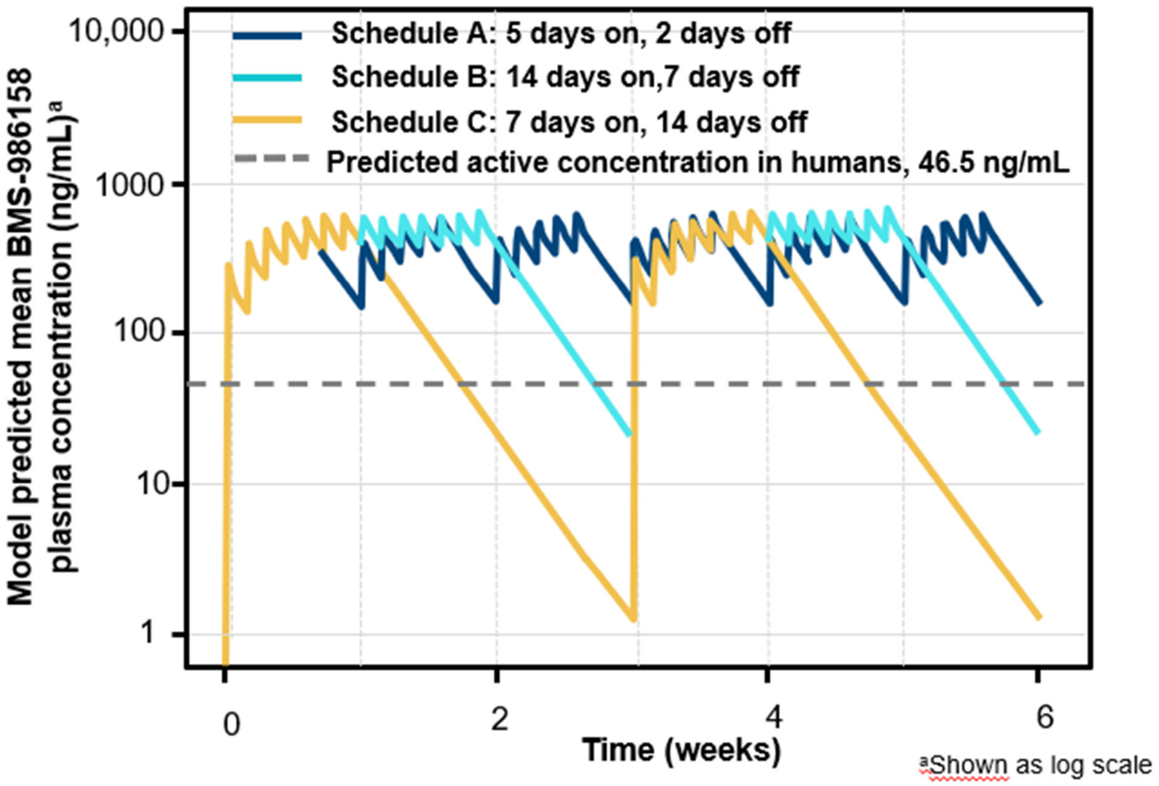

Figure S2. Predicted pharmacokinetic profile at BMS-986158 3-mg dose for each treatment schedule.

A)

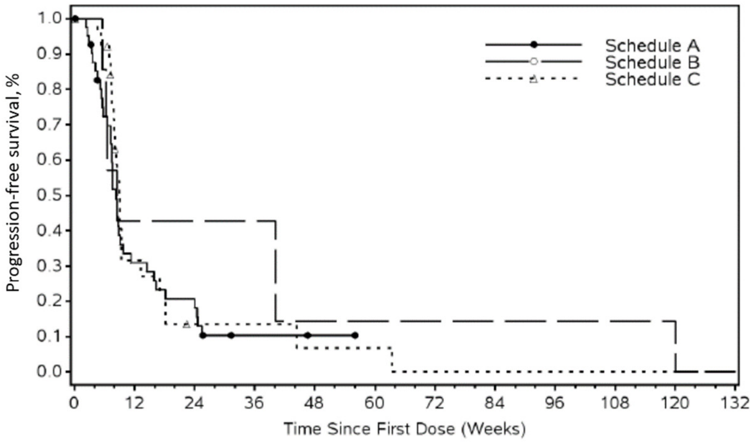

<u>Schedule</u>	<u>Number at risk</u>											
**A**	46	12	8	2	1	0	0	0	0	0	0	0
**B**	8	3	3	3	1	1	1	1	1	1	1	0
**C**	29	7	2	2	1	1	0	0	0	0	0	0

B)

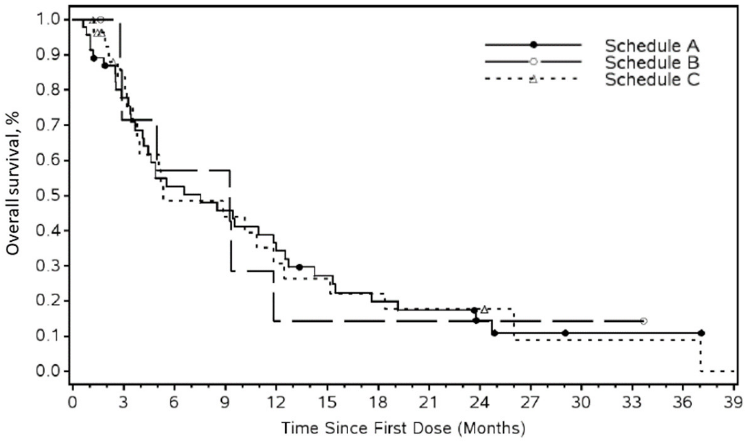

<u>Schedule</u>	<u>Number at risk</u>												
**A**	46	34	23	20	15	11	8	7	4	2	1	1	1	0
**B**	8	5	4	4	1	1	1	1	1	1	1	1	0	0
**C**	29	19	11	10	7	6	5	4	4	1	1	1	1	0

Figure S3. Kaplan-Meier survival curves by treatment schedule for A) PFS and B) OS.

A)

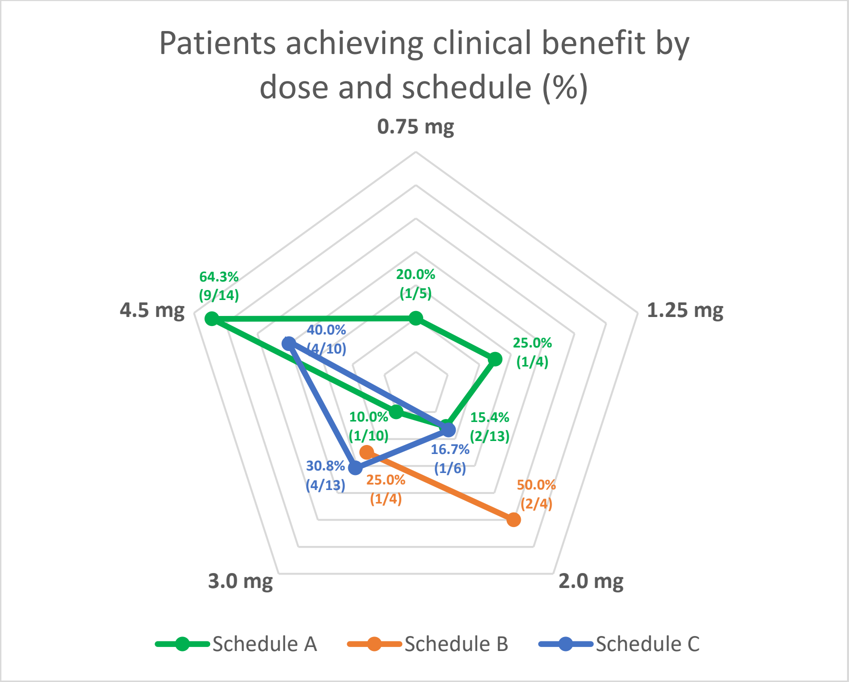

B)

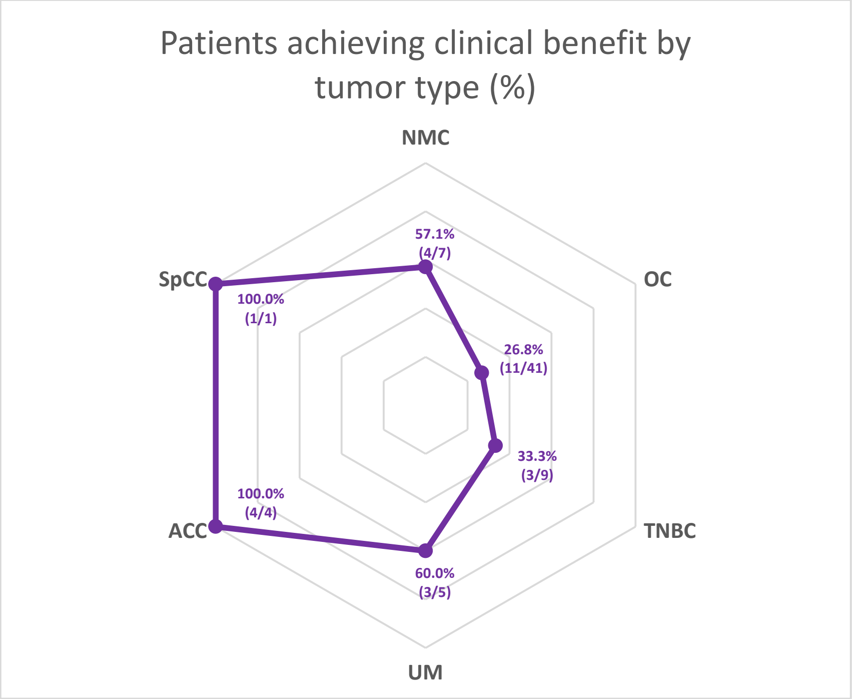

Figure S4. Patients who achieved clinical benefit (SD or better) by A) dose and schedule and B) tumor type.

Table S1. Pharmacokinetics of BMS-986158 and its metabolite at single dose by treatment schedule.

**Pharmacokinetic parameter**	**Assigned to the cohort receiving:**									
	**Schedule A**					**Schedule B**		**Schedule C**		
	**0.75 mg n = 5**	**1.25 mg n = 3**	**2.0 mg n = 9**	**3.0 mg n = 10**	**4.5 mg n = 10**	**2.0 mg n = 1**	**3.0 mg n = 4**	**2.0 mg n = 6**	**3.0 mg n = 11**	**4.5 mg n = 7**
	**BMS-986158**									
C_{max} (ng/mL), GM (%CV)	68.8 (23)	175 (36)	260 (20)	328 (36)	478 (24)	207	481 (25)	295 (29)	370 (22)	567 (24)
T_{max} (h), median (range)	4.00 (2.00–4.02)	1.00 (1.00–2.00)	2.00 (1.00–4.03)	2.00 (0.98–6.15)	2.04 (1.00–4.03)	1.00	1.03 (1.00–4.03)	1.00 (0.50–2.03)	1.00 (0.50–2.02)	2.02 (1.00–4.00)
$AUC_{(0–24)}$ (h×ng/mL), GM (%CV)	1027 (16)	2309 (13)	3610 (22)	4942 (43)	6786 (24)	2358	6921 (28)	3660 (27)	4468 (29)	7418 (43)
$AUC_{(0–T)}$ (h×ng/mL), GM (%CV)	2150 (15)	6372 (14)	8564 (48)	10,452 (50)	19,124 (46)	5202	28,852 (41)	10,931 (43)	11,493 (54)	17,220 (84)
Effective $T_{1/2}$ (h), mean (SD)	33.7 (1.4)[a]	48.7 (6.7)	41.4 (18.2)[a]	33.3 (7.6)[b]	48.3 (13.4)[c]	44.2	54.0[d]	64.6 (17.5)	48.2 (23.8)[e]	35.7 (17.8)[f]
Vz/F (L), GM (%CV)	14.7 (21)[a]	12.5 (18)	14.6 (15)[a]	12.8 (25)[b]	15.7 (29)[c]	23.2	15.3[d]	13.9 (29)	15.6 (28)[e]	11.6 (17)[f]
	**Metabolite of BMS-986158**									
C_{max} (ng/mL), GM (%CV)	5.0 (30)	10.0 (29)	16.9 (32)[g]	22.5 (38)[c]	30.0 (41)	13.6	26.3 (63)	21.1 (35)	19.4 (44)	45.5 (32)
MR C_{max}, GM (%CV)	0.07 (14)	0.06 (9)	0.07 (30)[g]	0.07 (34)[c]	0.06 (45)	0.07	0.06 (42)	0.07 (48)	0.05 (34)	0.08 (25)
T_{max} (h), median (range)	24.0 (2.6–24.2)	2.0 (2.0–24.0)	5.1 (2.0–72.0)[g]	23.9 (1.0–48.0)[c]	15.1 (1.0–48.0)	1.00	1.52 (1.0–47.4)	14.9 (1.0–71.5)	2.02 (1.0–48.0)	6.27 (1.0–45.6)
MR $AUC_{(0–24)}$ (h×ng/mL), GM (%CV)	0.10 (29)	0.08 (7)	0.08 (24)[g]	0.09 (44)[c]	0.08 (40)	0.08	0.06 (33)	0.10 (18)	0.08 (35)	0.10 (41)
MR $AUC_{(0–T)}$ (h×ng/mL), GM (%CV)	0.15 (33)	0.12 (29)	0.12 (21)[g]	0.14 (38)[c]	0.14 (37)	0.08	0.10 (28)	0.17 (35)	0.11 (41)	0.15 (48)

[a]n = 4; [b]n = 7; [c]n = 9; [d]n = 1; [e]n = 10; [f]n = 5; [g]n = 8.

$AUC_{(0–24)}$, area under the plasma concentration-time curve from time 0 to time 24 hours post dose; $AUC_{(0–T)}$, area under the plasma concentration-time curve from time 0 to time of the last quantifiable concentration; CL/F, apparent total body clearance; C_{max},

maximum observed plasma concentration; %CV, coefficient of variation; GM, geometric mean; MR, metabolite-to-parent; N/A, not available; $T_{1/2}$, half-life; T_{max}, time to C_{max}; Vz/F, apparent volume of distribution.

Table S2. Baseline treatment assignments for BMS-986158 monotherapy and tumor type [a].

Characteristic	Patients who achieved clinical benefit, n/N (%)[a]
**All safety population**	26/83 (31.3)
**Schedule A, all doses**	14/46 (30.4)
**0.75 mg**	1/5 (20.0)
**1.25 mg**	1/4 (25.0)
**2.0 mg**	2/13 (15.4)
**3.0 mg**	1/10 (10.0)
**4.5 mg**	9/14 (64.3)
**Schedule B, all doses**	3/8 (37.5)
**2.0 mg**	2/4 (50.0)
**3.0 mg**	1/4 (25.0)
**Schedule C, all doses**	9/29 (31.0)
**2.0 mg**	1/6 (16.7)
**3.0 mg**	4/13 (30.8)
**4.5 mg**	4/10 (40.0)
**Tumor type, n (%)**	
**NUT carcinoma**	4/7 (57.1)
**Ovarian cancer**	11/41 (26.8)
**TNBC**	3/9 (33.3)
**UM**	3/5 (60.0)
**ACC**	4/4 (100.0)
**SpCC**	1/1 (100.0)

[a]Percentage of patients with SD or better calculated as number of patients with SD or better divided by number of patients in the safety population with the same characteristic multiplied by 100.

ACC, adenoid cystic carcinoma; NUT, nuclear protein in testis; QD, once daily; SpCC, spindle cell carcinoma; TNBC, triple-negative breast cancer, UM, uveal melanoma.
